# Supplementary material for: Subjective and objective measures of visual awareness converge
Source: PLoS One. 2023 Oct 3;18(10):e0292438. doi: 10.1371/journal.pone.0292438 (PMC10547206; doi:10.1371/journal.pone.0292438)
Supplement: S1 File — (PDF) [file pone.0292438.s001.pdf]

Supplement

Subjective and objective measures of visual awareness converge

Markus Kiefer, Verena Frühauf and Thomas Kammer

Department of Psychiatry, Ulm University, Germany

Table S1: Thresholds - Results from rmANOVA

Factor	df	F	p	η_p^2
TASK	1,19	193.6	<.001	.911
CONTRAST	1,19	274.4	<.001	.935
MODE	1,19	0.78	.388	.039
TASK x CONTRAST	1,19	12.5	.002	.397
TASK x MODE	1,19	0.11	.740	.006
CONTRAST x MODE	1,19	0.96	.339	.048
TASK x CONTRAST x MODE	1,19	1.55	.227	.076

Table S2: Width - Results from rmANOVA

Factor	df	F	p	η_p^2
TASK	1,19	17.6	<.001	.481
CONTRAST	1,19	2.36	.141	.111
MODE	1,19	49.0	<.001	.721
TASK x CONTRAST	1,19	3.27	.086	.146
TASK x MODE	1,19	0.39	.540	.02
CONTRAST x MODE	1,19	20.4	<.001	.518
TASK x CONTRAST x MODE	1,19	2.22	.153	.104

Analysis per Interval

We separated data according to the interval where the target stimulus was presented during temporal 2-AFC: interval 1 or interval 2. Psychometric functions were fitted to the accuracy distribution (objective response) as well as to the PAS rating distribution, similar to the main analysis. For the detection task this was possible in 16 subjects, whereas for the discrimination task in all 20 subjects we obtained a psychometric function. Placement of the target in interval 1 or interval 2 was at random; mean occurrence in interval 1 was 37.5 ± 4.5, range 29 - 49, and in interval 2 mean occurrence was 38.5 ± 4.5, range 27 – 47. Data from both tasks were separately subjected to an rmANOVA with the within-factors INTERVAL, CONTRAST, and MODE (see Tables S3, S4 and Figure S1 for detailed results). For both tasks, detection and discrimination, a main effect was found for CONTRAST (statistical values in the tables). In both tasks the high contrast runs yielded lower thresholds. The critical interaction INTERVAL x MODE was significant in both tasks. For the objective responses, estimated thresholds were lower in interval 1 compared to interval 2 (post-hoc comparison: detection $p = .018$, discrimination $p < .001$). Thresholds for subjective responses did not differ in the detection task, whereas in the discrimination task thresholds in interval 2 were lower compared to interval 1 (post-hoc comparison: $p < .001$). This analysis demonstrates that in objective responses observers seem to prefer interval 1, whereas in subjective responses no consistent pattern was observed. At least in case of the detection task, estimated thresholds to subjective responses were not different in interval 1 and interval 2. The most plausible explanation for the difference in objective and subjective responses is that in case of invisibility of the target in the objective task subjects tend to guess that the

target is presented in interval 1. This did not happen in the subjective ratings in the detection task. In the discrimination task, thresholds of subjective ratings were even lower in interval 2 compared to interval 1. Please notice, that in the concept of temporal 2-AFC tasks a separate analysis of responses to interval 1 and interval 2 is not intended. Analyzing both intervals together, in case of uncertainty responses will be at random.

Table S3: Thresholds per interval in the detection task. Results from rmANOVA

Factor	df	F	p	η_p^2
INTERVAL	1,15	1.18	.29	.073
CONTRAST	1,15	50.0	<.001	.769
MODE	1,15	0.0018	.967	.0001
INTERVAL x CONTRAST	1,15	4.21	.058	.219
INTERVAL x MODE	1,15	4.70	.0467	.239
CONTRAST x MODE	1,15	1.03	.326	.0064
INTERVAL x CONTRAST x MODE	1,15	0.171	.685	.0112

Table S4: Thresholds per interval in the discrimination task. Results from rmANOVA

Factor	df	F	p	η_p^2
INTERVAL	1,19	0.05	.83	.0025
CONTRAST	1,19	121.1	<.001	.864
MODE	1,19	3.70	.070	.162
INTERVAL x CONTRAST	1,19	9.66	.0058	.337
INTERVAL x MODE	1,19	52.6	<.001	.735
CONTRAST x MODE	1,19	2.83	.108	.130
INTERVAL x CONTRAST x MODE	1,19	4.77	.0418	.201

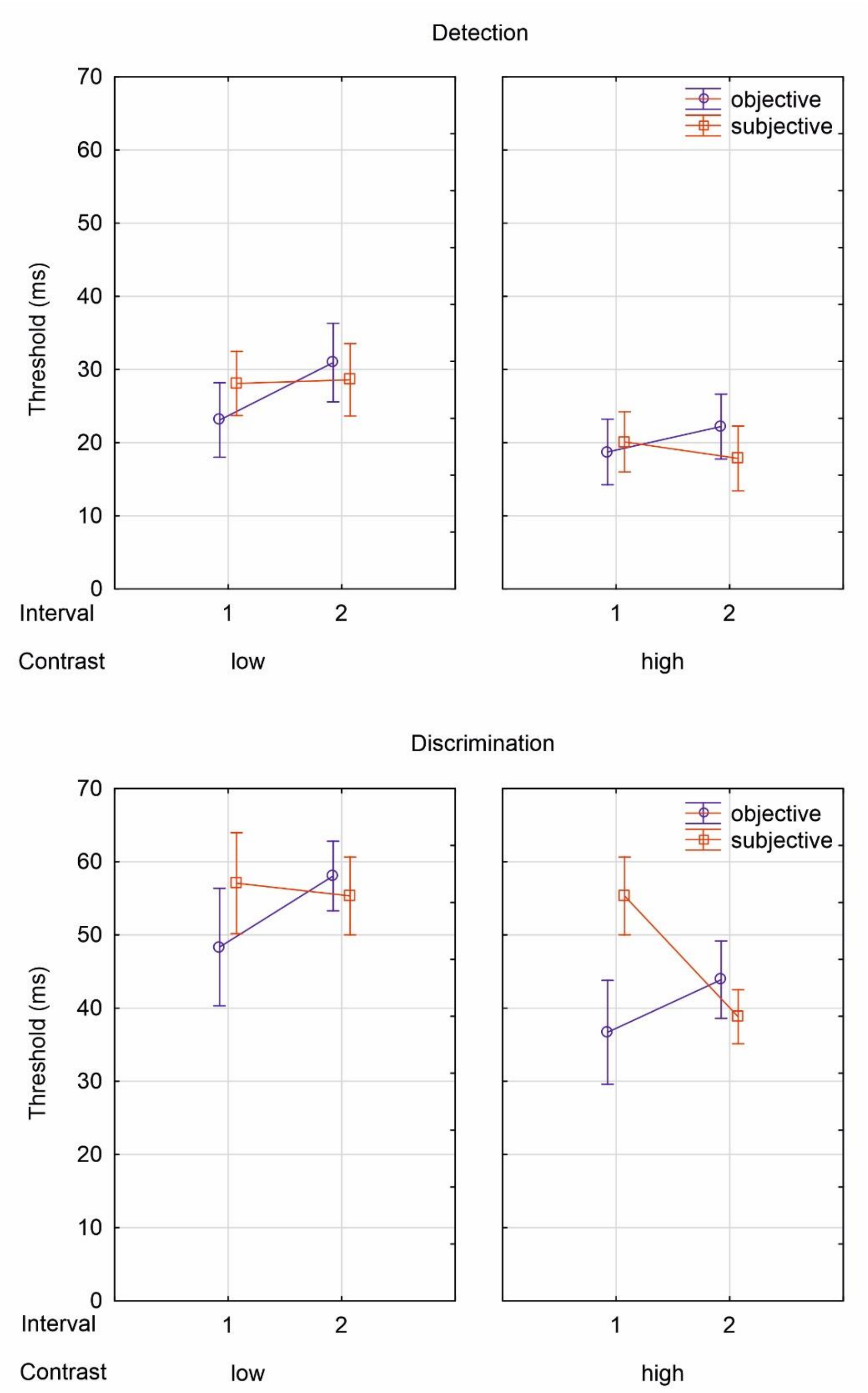

Figure S5: Mean objective and subjective thresholds per interval in the detection and discrimination tasks as a function of contrast. Error bars depict 95% confidence intervals (within design). On the abscissa, the different conditions are shown.
