# Supplementary material for: Subjective and objective measures of visual awareness converge
Source: PLoS One. 2023 Oct 3;18(10):e0292438. doi: 10.1371/journal.pone.0292438 (PMC10547206; doi:10.1371/journal.pone.0292438)
Supplement: Attachment — Submitted filename: ResponseLetter_rev2.docx [file pone.0292438.s003.docx]

**Responses to the Editor and to the Reviewers**

We would like to thank the editor and the reviewers for their positive evaluation of our manuscript and the thoughtful and stimulating comments. We are happy to see that we successfully addressed all major concerns. Reviewer 1 was fully satisfied with our revisions, reviewer 2 had some minor points, while the editor made further suggestions for improvements. When preparing the revision, we have carefully taken all comments into account and have changed the manuscript accordingly. All changes are marked in the manuscript in yellow color.

**Editor**

*(-1-) overly lengthy discussion*

*Although the manuscript is generally well-written and concise, this ceases to be the case at the onset of the discussion section. While I find the discussion to be insightful and thoughtfully debated, it is somewhat lengthy, and overly focused on technical aspects, in my estimation. Consequently, my recommendation would be to revise and streamline the discussion section to make it more succinct.*

Response: Thank you for evaluating our manuscript as well-written and concise. We agree with you that the discussion is somewhat lengthy and focused on technical aspects at places. Please note that both reviewers requested to discuss these technical issues related to the nature of the 2-AFC task, fitting of psychometric functions, interpretation of the parameters of the psychometric function, etc. in detail. However, we added the discussion of these technical issues to not only satisfy our reviewers’ concerns, but also felt that a clarification of these issues, which are frequently raised, but rarely publicly addressed by researchers, would be insightful for the readership, at least for those with some interest in methodological aspects of psychophysics or psychometrics. We therefore decided to keep these technical issues, although we are aware of the broad interdisciplinary readership of PLOS One. In order to accommodate your suggestion to streamline the discussion section, we removed redundancies or reiterations at several places (text deleted between lines 716-717, and between lines 732-747). We hope that these changes will make the discussion section more concise.

*(-2-) objective and subjective measures are different processes*

*While I am not an expert in the field, it appears to me that the question of whether objective and subjective measures represent different processes could potentially be addressed a priori through meticulous analysis of the necessary components and characteristics of the process in question. To name an example of what I mean, I suggest some specific literature that did such an analysis of the relationship between introspective and performative outcomes (doi:10.3389/fpsyg.2022.867978, chap. 4.3., 4.4, 4.5). Empirical verification in this context may be quite challenging, given that it relies on the design of the experiment and the specific factors manipulated. In the case of a temporal 2-AFC task, for instance, there are crucial factors that naturally influence the awareness threshold. At this juncture, I would expect a more comprehensive analytical exploration into the matter.*

Response: Thank you for suggesting a more comprehensive analytical exploration of commonalities and differences of objective and subjective measures of awareness and for drawing our attention to the interesting review article on cognitive restauration by Schumann and colleagues, which is now cited in our manuscript. In the previous version of the manuscript, we have already provided detailed analyses regarding the processes underlying objective and subjective measures, possible commonalities and differences and associated measurement problems. In fact, the analytical cognitive explorations provided in the manuscript lead to the three possible scenarios regarding the relation between objective and subjective measures of awareness described at the end of the introduction section (independence of objective vs. subjective measures, relation, but constant lag, zero lag). Perhaps, these analytical cognitive explorations were not particular salient in the previous version of our manuscript, because they were provided at various places in the introduction section. As a first measure, we moved the definitions of access vs. phenomenal consciousness from the end to the manuscript to the beginning of the introduction section (p. 4, lines 68-73). Furthermore, in order to make the connection between the three scenarios of a relation between objective and subjective measures of awareness and the analytical cognitive explorations in the preceding paragraphs of the manuscript more salient, we shortly repeat the key findings or theoretical arguments related to a given scenario at the end of the introduction section (p. 11, lines 247-263): “With regard to the relation between objective performance and subjective PAS ratings measures, depending on the precise theoretical stance, three alternative hypothetical scenarios are possible: Firstly, thresholds derived from objective and subjective measures of awareness could be unrelated across tasks and contrasts, because these measures might capture qualitatively different forms of information or representations [31,32] linked to access vs. phenomenal consciousness [1,43]. Objective and subjective measures may also partially depend on different types processes [for a discussion, also see 44]: For instance, only subjective, but not objective measures might depend on second-order meta-cognitive processes, which evaluate the representation [33]. Secondly, objective and subjective thresholds could exhibit a comparable pattern as a function of task and contrast, but subjective thresholds would be temporally delayed by a constant lag. This lag may arise because above-threshold objective performance might be partially based on fast unconscious processing, while above-threshold subjective ratings may require longer lasting visual consolidation giving rise to a specific phenomenal experience [10,18,28]. Thirdly, PAS ratings and accuracy of task performance could comparably reflect the phenomenal content of visual awareness. As a consequence, thresholds should be similar for subjective and objective measures, exhibiting a zero-lag.”

*(-3-) subjective and objective measures within same trial*

*Your arguments to acknowledge a potential criticism—that the convergence of measures might be due to the fact that both subjective and objective measurements were collected within the same trial, could be reconsidered. I would clearly agree with the authors that pointing out that previous research, which also collected data within the same trial, did not find such convergence, in and in this way, the present research corroborates previous findings, which could be an argument that no more data collection would be needed to corroborate the present finding. In my layman perspective, however, I feel that this aspect of design may indeed by crucial here. I name an example from another field: when introspecting inattention in reaction-time series (see my aforementioned suggested review paper, around chap. 4.1 to 4.5), the self-ratings are typically given as probe trials as performance is sensitive to the frequency of "asking introspectively". Maybe it is more than to be in line with the other findings, but maybe you have some ideas of design features could be improved in future studies to - in my layman view - be better.*

Response: Thank you again for drawing our attention to the review paper on cognitive restauration by Schumann and colleagues, which discusses possible interactions between objective performance and introspection. We entirely agree that such interactions can in principle take place. In fact, we have already investigated such interactions in a previous study (Kiefer, M., Harpaintner, M., Rohr, M., & Wentura, D. (2023). Assessing subjective prime awareness on a trial-by-trial basis interferes with masked semantic priming effects. Journal of Experimental Psychology: Learning, Memory, and Cognition, 49(2), 269-283. doi: 10.1037/xlm0001228). However, with regard to the present study, we do not see how such interactions could induce a common threshold of awareness. Nevertheless, we added a short discussion of within-trial interactions including a citation of the Schumann et al. paper (p. 25, lines 574-579). Please note that in the previous revision round in response to reviewer 2 we already provided suggestions for design changes, in order to systematically assess such interactions. In the new discussion paragraph, we foreshadow these suggested design changes, which include collection of subjective and objective measures in different blocks of trials: ” Although introduction of subjective ratings can in principle influence performance in the same [52] or in the next trial [44], we do not see how interactions between subjective and objective awareness tasks could induce a convergence of thresholds. Nevertheless, to specifically examine cross-task interactions, future studies could collect subjective and objective awareness measures in different trials (for a further discussion, see below).”

*(-4-) slopes for easy and complex tasks*

*One crucial observation the authors point on is that shallower slopes (i.e., wider widths) were associated with more complex tasks (e.g., discrimination vs. detection). This result supports the notion that the transition from unawareness to awareness may differ based on the complexity of the perceptual features being evaluated. This is an absolutely interesting and in my view crucial theoretical argument that merit stronger emphasis, at least I feel it so by impulse. My immediate association is a recent work of Cao et al. (doi:10.1007/s00221-020-05861-4) who argued in the same direction supporting the author's theoretical proposal, namely that even the complexity of the rather motoric task or movement has a fundamentally altering effect on individual's introspective judgment. I suggest giving this theoretical argument more elaboration in the final revision.*

Response: Thank you for recommending the paper by Cao and colleagues on action binding and the time course of the perceived action depending on action complexity. It is indeed intriguing that a similar more gradual transition from unawareness to awareness for complex vs. simple features has been suggested for somatosensory features associated with actions. As this editor mentions in his point 1 that the discussion section is already quite long, we added just two short sentences to highlight this aspect (pp. 26-27, lines 621-625): “Interestingly, a similar more gradual transition from unawareness to awareness for complex vs. simple features has been suggested for somatosensory features associated with actions [55]. Possibly, a more gradual emergence of awareness for complex features might be a functional principle also in other domains than vision.”

*(-5-) take home message*

*Although I am not an expert, I must express some reservations regarding the final conclusions. While there is no doubt that the findings are the result of an outstanding empirical study, the theoretical conclusions—namely, that the study challenges the traditional dichotomy between access and phenomenal consciousness—merit scrutiny. My scepticism arises from the belief that the design methodology (typically accepted within the field) may not be sufficiently critical to reveal these rather subtle differences, and this exactly is what future research should be more aware of.*

Response: Please note that we have never argued that our study challenges the traditional dichotomy between access and phenomenal consciousness. As these types of consciousness essentially differ with regard to their perspective (third vs. first person perspective), this dichotomy cannot be resolved, even if objective and subjective measures of awareness converge. We have only argued that access and phenomenal consciousness possibly rely on the same representations. As this statement might be misleading and could evoke the impression that we question the dichotomy of access and phenomenal consciousness, we have deleted in the discussion section all statements (p. 31, lines 732-747), which could evoke connotations in this direction. This has the further advantage that the final conclusion section is much shorter now in line with the editor’s point 1. It now reads (p. 31, lines 741-747): In conclusion, using a temporal 2-AFC task we found a comparable pattern of thresholds across tasks and contrasts for objective and subjective measurements of awareness. This finding suggests that objective performance measures based on accuracy and subjective ratings of the visual experience can provide similar information on the feature-content of a percept [see also, 51]. The observed similarity of thresholds validates both psychophysical and subjective approaches to awareness as converging and thus most likely veridical measures of awareness.

**Reviewer 1**

All comments have been addressed

Response: We are glad to see that we have addressed this reviewer’s points satisfactorily.

**Reviewer 2**

Overall, I think the authors have revised this manuscript thoroughly and carefully. They have responded extensively to all of my previous comments. I think the additional explanations in the methods section help clarify the procedure. I like the newly added Fig 3, as it is very illustrative of the effects. I am still not so strongly convinced by the null effect on which the main interpretation of the results rests, as the final sample was rather small (N=20) and the power analysis was based on the assumption of a rather large effect size. In addition, the null effect is very unexpected based on the results of previous studies and thus stands quite isolated in the literature. The explanations of the authors for this divergence are plausible but purely speculative at this point. The novel analysis on the order effects does not exactly help, either, as the pattern of thresholds from the subjective ratings do not follow the objective thresholds, but given the small trial numbers I agree that this should not be strongly interpreted (and it is also questionable if the order bias in the objective thresholds would be accessible to subjective report). So I am still a bit ambiguous of this manuscript - on the one hand I find the approach and the results interesting (and of course I am not opposed to publishing null results). On the other hand, I feel that the theoretical contribution of this manuscript would be so much stronger if the null results were replicated in a second, more highly-powered, experiment; or if the authors attempted to empirically explore the reasons for the divergence of their and previous results, so publishing the work at this stage feels a bit like a missed opportunity for a much higher-impact contribution.

Response: We thank this reviewer for acknowledging our effort in addressing their concerns and in improving our manuscript. We feel honored that this reviewer acknowledges the theoretical importance and empirical soundness of our study. It is true that replication of the present findings and exploration of suggested reasons for divergent findings are important. We would like to mention at this place that somewhat related to the present work, a recent study using constant mask stimulus SOAs, just published a few weeks ago, found comparable objective and subjective d’ sensitivity measures. Hence, our observation of convergence of objective and subjective measures is not so isolated any more. We now mention this study in the revised manuscript on p. 24, lines 545-549. Given that so many new issues could and should be addressed in future studies, we felt that adding one new experiment to this manuscript exploring one question would still appear somewhat unsatisfactory because other 3 or 4 questions would have still not been addressed. We certainly share the interest with this reviewer to get these new questions answered, but think it is more appropriate to do this in a future publication, given that the present work is already fairly complex

I spotted a few typos and other minor points:
- P. 3: …on a four-point rating scales…-> scale
- P. 16: … we did not sufficiently collected data…-> collect a sufficient number of data points
- P. 16: to empirically confirm guessing rate of 2AFC -> a guessing rate for the 2AFC task
- P. 19: for low contrast condition compared to high contrast condition -> the low, the high
- P. 20: compared to discrimination task -> to the discrimination task
- P. 24: subjective ratings threshold were -> thresholds
- P. 25: we analyzing thresholds -> analyzed
- P. 30: could also test, whether the thresholds would still converge, if the … -> remove commata
- Fig 2: shouldn’t the x-axis on the first column be labeled “trial” instead of “run #”

Response: Thank you for highlighting these typos and minor points. The manuscript has been changed as suggested, including the label on the abscissa in the first column of Fig 2 (“trial”).
